# Environmentally responsible human genomic data governance: points for consideration

**DOI:** 10.1038/s41525-026-00614-8

**Published:** 2026-09-03

**Authors:** Narcyz Ghinea, Gabrielle Samuel, Wendy Xin, Emma Bonser, Robert Cook-Deegan, Monica Ferrie, Christopher Gyngell, Margaret Otlowski, Alan Petersen, Bridget Pratt, Susie Roczo-Farkas, Ainsley J. Newson

**Affiliations:** 1 Sydney Health Ethics Faculty of Medicine and Health, Sydney School of Public Health, University of Sydney, Sydney, NSW Australia; 2 Department of Global Health and Social Medicine, King’s College London, London, UK; 3 Genetic Alliance Australia, Darlinghurst, NSW Australia; 4 Arizona State University, Washington, DC USA; 5 Genetic Support Network Victoria, Parkville, VIC Australia; 6 Department of Paediatrics, The University of Melbourne, Melbourne, VIC Australia; 7 Biomedical Ethics, Murdoch Children’s Research Institute, Melbourne, VIC Australia; 8 Melbourne Law School, The University of Melbourne, Parkville, VIC Australia; 9 Faculty of Law, Centre for Law and Genetics, University of Tasmania, Hobart, TAS Australia; 10 School of Social Sciences, Monash University, Clayton, VIC Australia; 11 Queensland Bioethics Centre, Australian Catholic University, Brisbane, QLD Australia; 12 School of Population and Global Health, University of Melbourne, Melbourne, VIC Australia

**Keywords:** Environmental sciences, Environmental social sciences, Environmental studies, Genetics, Geography, Geography, Scientific community, Social sciences

## Abstract

We introduce five points for integrating environmental ethics into human genomic data governance: (i) recognizing the ethical imperative to consider environmental impacts of human genomic data; (ii) fostering collective responsibility for environmental harms; (iii) prospectively assessing benefits and harms; (iv) anticipating barriers to integration of environmental ethics into genomic data governance; and (v) meaningfully engaging all interest-holders. These points will be useful to all involved in the genomic data ecosystem.

## Introduction

Human genomic research and genomic medicine are rapidly expanding, bringing significant data storage requirements. With genomics initiatives generating data from increasing numbers of people globally, the potential for further genomic data growth is extensive. Drawing on one current national genomics initiative, Genomics England currently sequences 80,000 patient genomes per year. This data and its related outputs require 100 petabytes of storage space^1^, more than the annual data generated by the world’s largest science instrument, the Large Hadron Collider (~90 petabytes of data per year)^2^.

These figures only consider the storage of human genomic data, not its generation, processing, analysis or sharing. Utilizing human genomic data will have environmental impacts, including impacts from energy consumption, water use, land use and carbon emissions due to energy-intensive laboratories and large-scale digital infrastructures^3,4^. Supply chains and disposal of equipment and genomic testing consumables are also relevant. When we consider the whole genomic data lifecycle—which also includes mining of raw materials needed for manufacturing digital hardware and sequencing tools to sustain genomics—it is clear that the environmental impact of genomic data cannot be ignored^5^.

Even though we cannot discern the total amount of human genomic data currently stored globally, nor the total being generated each year, we can be confident that it is substantial. The environmental impacts of genomics will continue to accelerate as whole genome sequencing becomes more accessible a in clinical practice and research^6,7^. The environmental impact of genomic data storage, and how we might implement it in an environmentally sustainable and responsible way, is therefore important, particularly considering the potential for exponential growth in coming years as genomics is mainstreamed in healthcare.

Alongside the expansion in human genomic data generation, processing, use, storage and sharing, novel genomic data governance frameworks are being developed to address socio-ethical and legal concerns around aspects such as privacy, security and consent^8^. Broadly, a genomic data governance framework is a set of agreed principles and values used to define the purposes and safeguards for access to, as well as use, sharing, and stewardship of human genomic data. Environmental considerations have received scant attention in such governance to date^9^.

Environmental ethics considers the moral implications of human activity with respect to the natural world^10^, and we contend that environmental (bio)ethics has an important role to play in genomic data governance^11,12^. In this commentary, we introduce five high-level points for consideration regarding environmental ethics and human genomic data governance. These points were collaboratively developed at an interdisciplinary workshop held in August 2025, under the auspices of the LINEAGE study (https://lineagestudy.org.au/).

## Points to consider in environmentally responsible human genomic data governance

### (i) Recognize the ethical imperative to consider environmental impacts of human genomic data

Whether we commit to an eco-centric or anthropocentric worldview, the need to protect the environment is imperative. Those holding anthropocentric views recognize that human flourishing cannot occur without a flourishing natural world^13^. Eco-centrists agree that the environment has both instrumental value for humanity and intrinsic value^14^. Regardless of the perspective one aligns with, environmental harms stemming from human activities need to be assessed, both generally and regarding genomic data governance, for the well-being of both the natural planet and humanity. While there may be circumstances in which environmental harm is justified to advance human interests, any exception requires justification, particularly when benefits remain largely promissory, contested, or where there are equally effective greener alternatives available^15,16^.

The imperative to consider the environmental harms of human genomic data should not only sit with those who develop technological solutions, but with all who use such data. Greener energy sources, greener algorithms, improved digital waste management, and more energy-efficient data storage protocols and formats could mitigate some environmental impacts, but these benefits will only be realized if those working with human genomic data account for those impacts in their practice^12,17^. It is well recognized that greater system efficiencies can lead to more intensive utilization, which can perversely undermine the environmental benefits of any efficiency gains^18^. For instance, all technologies that enhance work productivity do not axiomatically lead to working fewer hours, but rather to work intensification. In the same way, making genomics greener in terms of energy efficiency and resource utilization will not *necessarily* reduce its environmental impacts but may lead to more intensive use.

Because the value proposition of health genomics remains largely promissory (especially for people without European ancestry), it is imperative to balance intensification of human genomic data generation, processing, storage, use, sharing and so on with being mindful of who and what will benefit. Greener technologies do not mitigate the need to consider our responsibilities for environmental harms arising from our decisions about human genomic data.

### (ii) Take collective responsibility for environmental harms of human genomic data

To translate environmental concerns arising from human genomic data into action, all who are generating, storing and processing such data need to take responsibility for adopting environmentally conscious processes, and funders and payers need to ensure this happens. Yet taking responsibility can be complicated by the many institutions and individuals involved throughout the genomic data lifecycle, including sequencing providers, researchers, healthcare professionals, laboratory technicians, hospitals, biobanks, funding bodies, governments, and private companies. How responsibility for environmental harm should be distributed among these interest-holders remains an open question.

One challenge to upholding responsibility is that interest-holders need both the power and knowledge to respond^19^. As an example, one area where empirical data would be very useful is in comparing the environmental impact of clinical whole genome sequencing (WGS) against more targeted testing. Some health services’ procurement contracts only allow ordering of WGS, even when other approaches might be sufficient, leading to the generation of large amounts of additional data. If it turns out that WGS is more environmentally harmful, then clinicians’ autonomy to act in an environmentally conscious way will be limited, because of external contingencies such as responsible procurement.

Taking collective responsibility for environmental harms of health genomic data is likely to occur only through concerted top-down and bottom-up efforts. This can be, and is being, facilitated through grassroots advocacy and initiatives such as the development of tools and resources to understand the environmental impacts of decisions^20^, green accreditation schemes to increase visibility and provide alternatives^21^, and environmental impact assessments by funders or ethics committees to encourage researchers to be aware of the environmental consequences of their activities^22^. However, more action is needed^9,12^.

To ensure buy-in from interest-holders, environmental considerations arising from human genomic data must be framed in terms of shared benefits. Environmental considerations need to be recognized as integral to sound, efficient, and cost-effective human genomic data governance, rather than merely an inconvenient add-on. Our current environmental crisis is a consequence of collective inaction. One solution for environmentally conscious human genomic data governance rests in collective action and responsibility. While we are in the nascent stages of determining specific responsibilities for environmental harms in human genomic data governance frameworks, it is important to raise awareness of the need to identify and address them. This includes considering how clinical necessity and ethical obligations (such as to those with rare conditions) balance against environmental considerations. In turn, these actions will encourage all parties with an interest in genomic governance to be more critical about the value of data being collected and stored^11^.

### (iii) Prospectively assess and weigh the benefits and harms of human genomic data generation

While human genomic data generation, use, and sharing can improve human health and well-being, its benefits are being disproportionately enjoyed by certain populations^23^. However, the environmental harms arising from the genomic data and research lifecycle are not limited to people who may benefit, but are borne by whole societies, particularly people living in low-resource settings. For example, informal workers in low-income countries processing toxic e-waste created by technology industries in high-income settings, or informal workers working in harsh conditions to mine rare earth and other minerals, which leaves behind toxic byproducts^24,25^. These workers and their families and communities are least likely to benefit. While the precise magnitude remains unknown, human genomic data are a significant form of big data that contributes to environmental harms.

The justifiable benefits and harms of human genomic data in the context of the current environmental crisis need to be accurately measured and critically considered. We should ensure that the human genomic data we collect, store and share yields fruitful results on an equitable basis, and that the risks of collecting and using this data do not outweigh likely benefits. Good practice in clinical care and genomics research should take account of environmental harms, along with other harms to which human genomic data contributes.

### (iv) Anticipate barriers to integrating environmental considerations in human genomic data governance

Socio-political, cultural and structural barriers can inhibit our capacity to address environmental harms from human genomics. One existing issue that could lead to additional data generation in parallel is that institutions may not share data due to commercial, consent, and privacy commitments, or concerns about intellectual property and profitability^26–28^. Left unaddressed, these will continue to lead to duplication of effort, and potentially greater environmental impacts. If greener practices are more expensive to implement, it will also undermine their adoption. Mitigating these issues will require global cooperation to develop and implement relevant policies (or components within existing governance) and practices. Addressing the environmental impacts of human genomic data need not compel data custodians to disclose commercially sensitive or private information. Rather, trusted intermediaries, legal safe harbors, and controlled data sharing models, such as those used by the Structural Genomics Consortium, offer adaptable precedents.

Great optimism about human genomics can overshadow environmental considerations. Some may deem the environmental impact of human genomics insignificant and not worthy of serious consideration given the potential to improve human health and well-being. Optimism around the capacity for green energy and greener technologies to mitigate environmental harms from data-intensive human genomics may also be seen to mitigate moral responsibility for the environment. These systemic and cultural barriers need to be anticipated and responded to in governance frameworks to enable human genomics infrastructure and practices to be more environmentally sustainable.

### (v) Meaningfully engage all interest-holders

Meaningful engagement with different interest-holders is essential to better understand the potentially different values and priorities and how these can shape how human genomic data are governed. Such engagement will help raise awareness of the importance of environmental considerations in human genomics, and support environmentally aware governance and decision-making. It could also invite deliberation on some of the trade-offs between environmental harms and potential benefits arising from human genomic data generation, use and sharing. While the benefits are easy to identify when they do emerge (e.g., the development of a new treatment for a rare condition), the environmental harms arising from human genomic data are often vague, insidious, and broad-ranging, but no less real. A lack of visibility or specificity does not ethically justify overlooking these harms.

## Conclusion

These points for consideration offer key elements for integrating environmental ethics considerations into human genomic data governance. While the environmental impact of health genomics should be taken seriously, addressing the five points above will require ethical trade-offs. The data-intensive nature of human genomics and healthcare more generally is significant, and its potential to harm the environment is substantial. As data-intensive technologies rapidly proliferate, so do the environmental implications. We need to rethink the value of human genomic data, recognizing the wider implications of its generation and use. This imperative should be reflected in governance frameworks.

## Data Availability

No datasets were generated or analyzed during the current study.
